# Antitumor *Trans* Platinum DNA Adducts: NMR and HPLC Study of the Interaction Between a *trans*-Pt Iminoether Complex and the Deoxy Decamer d(CCTCGCTCTC)·d(GAGAGCGAGG)

**DOI:** 10.1155/MBD.2000.23

**Published:** 2000

**Authors:** Bjørn Andersen, Nicola Margiotta, Mauro Coluccia, Giovanni Natile, Einar Sletten

**Affiliations:** 1 Department of Chemistry University of Bergen Allegt.41 Bergen N-5007 Norway; 2 Department of Pharmaceutical Chemistry University of Bari Via E. Orabona 4 Bari 1-70125 Italy; 3 Department of Biomedical Sciences and Human Oncology P.za Giulio Cesare 11 Bari 1-70124 Italy

## Abstract

The single-stranded oligonucleotide 5′-d(CCTCGCTCTC) (I) was reacted with the antitumor *trans* platinum iminoderivative *trans*-[PtCl_2_{*E*-HN = C(OMe)Me}_2_] (*trans-EE*) and subsequently annealed with its complementary strand 5′-d(GAGAGCGAGG) (II). The platinated duplex was characterized by 1D and 2D proton NMR spectroscopy at 600 MHz. In agreement with previous studies by different techniques *trans-EE* was found to form a monofunctional adduct with the duplex involving the guanine residue. The modification by *trans-EE* has been found to induce only minor local distortion in the duplex geometry. Two key crosspeaks observed in the NOESY map corresponding to a close contact between G5-H8 and the methoxy and the methyl group, respectively, enabled us to dock the *trans-EE* complex with the duplex by geometry optimization. The results support the idea that the antitumor activity of *trans-EE* is related to lesion of DNA fundamentally different from that of cisplatin. Unexpectedly, the NOESY spectra indicated that at the high NaCl concentration used (0.2 M) the duplex was found to undergo slow deplatination. This was subsequently proved by HPLC. In a separate experiment on platination of the single strand in a salt free environment the HPLC analysis showed that the monofunctional adduct was not deplatinated, however, after 24 hours, additidnal minor isomers were detected.


### ANTITUMOR *TRANS* PLATINUM DNA ADDUCTS: NMR AND HPLC STUDY OF THE INTERACTION BETWEEN A *trans*-Pt IMINOETHER COMPLEX AND THE DEOXY DECAMER d(CCTCGCTCTC)·d(GAGAGCGAGG)

Bjørn Andersen[1], Nicola Margiotta[2], Mauro Coluccia[3],

Giovanni Natile[2] and Einar Sletten*[1]

[1] Department of Chemistry, University of Bergen, Allegt.41, N-5007 Bergen, Norway
[2] Department of Pharmaceutical Chemistry, University of Bari,
Via E. Orabona 4, I-70125, Bari Italy
[3] Department of Biomedical Sciences and Human Oncology,
P.za Giulio Cesare 11, I-70124, Bari, Italy

**Abstract**

The single-stranded oligonucleotide 5′-d(CCTCGCTCTC) (I) was reacted with the antitumor *trans* platinum iminoderivative *trans*-[$PtCl_2${*E*-HN=C(OMe)Me}$_2$] (*trans-EE*) and subsequently annealed with its complementary strand 5′-d(GAGAGCGAGG) (II). The platinated duplex was characterized by 1D and 2D proton NMR spectroscopy at 600 MHz. In agreement with previous studies by different techniques *trans-EE* was found to form a monofunctional adduct with the duplex involving the guanine residue. The modification by *trans-EE* has been found to induce only minor local distortion in the duplex geometry. Two key crosspeaks observed in the NOESY map corresponding to a close contact between G5-H8 and the methoxy and the methyl group, respectively, enabled us to dock the *trans-EE* complex with the duplex by geometry optimization. The results support the idea that the antitumor activity of *trans-EE* is related to lesion of DNA fundamentally different from that of cisplatin. Unexpectedly, the NOESY spectra indicated that at the high NaCl concentration used (0.2 M) the duplex was found to undergo slow deplatination. This was subsequently proved by HPLC. In a separate experiment on platination of the single strand in a salt free environment the HPLC analysis showed that the monofunctional adduct was not deplatinated, however, after 24 hours, additional minor isomers were detected.

### Introduction

The treatment of several human tumors is based on platinum chemotherapy. The antitumor activity of cis-diamminedichloroplatinum(II) (*cis*-DDP), the most widely used metal-based anticancer drug, has been associated with formation of adducts with DNA (for recent reviews see [1] Lippert (ed.) Cisplatin). Since the *trans* isomer of *cis*-DDP is clinically ineffective it was assumed that a structure-activity relationship exists where only *cis* geometry is therapeutically active. However, recently it has been shown that several analogues of *trans*-DDP exhibit antitumor activity comparable to that of *cis*-DDP [2-5]. Of particular interest is a group of iminoether derivatives analogous to *trans*-DDP: *e.g.* *trans*-[$PtCl_2$(iminoether)$_2$] which was shown to be endowed with significant *in vivo* antitumor activity [3,6]. Surprisingly, these compounds were even more cytotoxic than their congener *cis*-[$PtCl_2$(iminoether)$_2$] [2].

In addition to the *cis* and *trans* geometry, platinum-iminoether complexes can have either *E* or *Z* configuration depending on the relative position of the alkoxy group and the N-bonded Pt with respect to the C=N double bond, and this represents another aspect to be considered for evaluation of structure-activity relationships.

Several papers [6-10] have dealt with the cytotoxity of platinum iminoether derivatives in tumor cells. *Trans*-[$PtCl_2${*E*-HN=C(OMe)Me}$_2$] (*trans-EE*) has shown an activity comparable to that of *cis*-DDP in the P338 leukaemia system and exerts antitumoral effects on Lewis lung carcinoma, thus representing a *trans* platinum drug active *in vivo* on both limphoproliferative and solid metastasising murine tumors. Furthermore, the DNA binding mode of *trans-EE* in a cell free medium has been characterized by several spectroscopic methods [11]. As a result of these studies it has been concluded that *trans-EE* forms kinetically stable monofunctional adducts at guanine residues in double-helical DNA. As a consequence *trans-EE* is assumed to modify DNA in a way different to clinically ineffective *trans*-DDP.

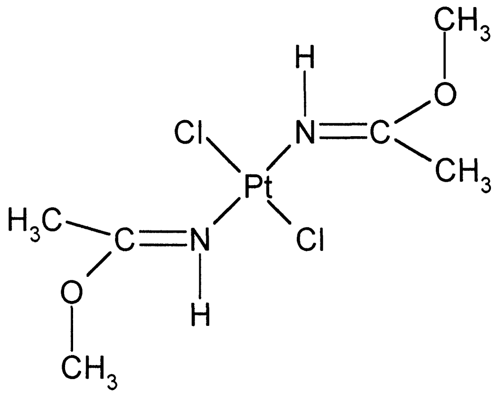

*Trans*-[$PtCl_2${*E*-HN=C(OMe)Me}$_2$] (*trans-EE*)

How can the subtle differences in DNA binding mode between *trans-EE* and *trans*-DDP explain the difference in antitumor activity? The nature of the non-leaving groups, i.e. the amine ligands in *trans*-DDP and the iminoether ligands in *trans*-EE, somehow play an important role and it has been speculated that in the latter case one of the drug - DNA interactions could not involve directly the metal ion but be mediated by the iminoether ligand. Such a type of interaction with DNA would place the platinum-iminoether complex at the junction between *cis*-DDP and non-metallic cytotoxic agents.

The effect of *cis-EE* and *trans-EE* binding upon the salt induced $B \rightarrow Z$ transition in DNA has been investigated in poly(dG-m$^5$dC) and poly(dG-dC) [11]. The authors used different techniques including circular dichroism spectroscopy, Raman spectroscopy and immunochemical assay. They concluded that the behavior of *trans-EE* was different from that of clinically ineffective *trans*-DDP, which hinders $B \rightarrow Z$ transition probably because of the interstrand cross-link formation [12].

In this paper, a detailed NMR spectroscopic analysis of the DNA duplex consisting of *trans-EE* platinated 5′-d(CCTCG*CTCTC) ($I_M$)and its complement 5′-d(GAGAGCGAGG) (II) is described. The platination reaction is also characterized by HPLC. The presented analysis reveals that the binding pattern of *trans-EE* is more complex than first anticipated involving pH and sequence-dependent isomerization reactions and deplatination if the NMR sample contains high NaCl concentration (0.2 M).

### Materials and Methods

*Sample preparation.*

The sequence was purchased from Oswell DNA Service as a HPLC purified compound. The reaction between *trans-EE* and the pyrimidine-rich strand 5'-d(CCTCGCTCTC) (I) containing a single G was performed using the aqua species [$PtCl(H_2O)${*E*-HN=C(OMe)Me}$_2$]$^+$ and the oligonucleotide in the molar ratio 4:1 at 25 °C and pH 6. The platinated oligonucleotide was purified by reverse phase HPLC using a Waters Symmetry C8 column and isocratic flow of 91 % $H_2O$ (0.02 M ($NH_4$)($CH_3COO$), pH 4.7) and 9% $CH_3CN$ [13]. The unique platination at the dG residue was verified by nonreactivity of dimethyl sulfate at this site [14], whereas the monofunctional nature of the adduct was verified by gel electrophoresis under denaturing conditions (24% polyacrylamide/8 M urea) of the products of reaction between the platinated oligonucleotide and thiourea, a sulfur-containing molecule which labilizes monofunctional trans platinum adducts from DNA [15]. The purified platinated oligonucleotide was then mixed with one equivalent of the complementary sequence, 5'-d(GAGAGCGAGG) (II). The platinated decamer NMR sample was 0.5 ml of 1.1 mM in duplex with 0.2 M NaCl, and pH at 5.2. No buffer was added. The sample of the unplatinated duplex was dissolved in 0.5 ml 90 $H_2$0/10 $D_2O$, 0.2 M NaCl, pH 5.5 and final concentration in duplex 3.0 mM. $^1$H NMR spectra were recorded in $H_2O$ and subsequently in $D_2O$ of freeze-dried samples.

*NMR Spectroscopy.*

The $^1$H NMR experiments were performed on a Bruker DRX 600 instrument, operating at 600 MHz for $^1$H NMR spectroscopy. In order to avoid melting of the duplexes and to obtain reasonable resolution all experiments were carried out at 295 K. 1D $^1$H spectra were collected with a total of 32K complex points and 64 transients. For all 1D and 2D spectra the spectral width were

6000 Hz (in $D_2O$) or 13200 Hz (in $H_2O$), and a relaxation delay of 2 s was used. The 3-9-19 WATERGATE pulse sequence was used for water suppression [16]. The $^1H$ NOESY spectra were recorded in the pure absorption mode with quadrature detection using the States-TPPI acquisition scheme [17]. A total of 2048 complex points in $t_2$ were collected for each of 512 $t_1$ increments and 80 transients were averaged for each increment. 2D NOESY spectra were acquired at two different mixing times (200 and 300 ms) in $D_2O$ and in $H_2O$. The FID's were processed by linear prediction routines increasing the time domain to 4K, which significantly improves the signal-to-noise in the NOESY map [18]. The NMR data were processed on a Silicon Graphics INDY workstation using the program FELIX (Biosym) [19] and on a 133 MHz Pentium PC using 1D and 2D WIN-NMR (Bruker) [20]. The one dimensional $^1H$ FID's were multiplied by an exponential window function prior to Fourier transformation. Typically, 1 Hz was added to the line widths. No baseline correction was needed. The assignments of two-dimensional NOESY's were performed on resolution enhanced spectra using a Gaussian function. The proton spectra were referenced to the HDO-resonance at 4.81 ppm (295 K).

*HPLC analysis.*

The HPLC analysis of the platinated and the unplatinated decamer, and the platination reaction analysis were performed on a Waters 626 LC instrument using Millenium 32 software [21]. A MonoQ HR 10/10 (Pharmacia Biotech) column was used with a 0.3 to 1.0 M NaCl gradient, containing 10 mM NaOH. A platination reaction between the single strand d(CCTCGCTCTC) (I) and excess of *trans-EE* was carried out at pH 2.5, to insure protonated cytosine and thymine, in $H_2O$ and at room temperature. The reaction was monitored by HPLC randomly during the first 48 hours after mixing the single strand and *trans-EE*.

*Molecular modeling*

Initially 500 cycles of energy minimization followed by 20ps of molecular dynamics calculations at constant temperature of 300 K were performed. The Verlet Leapfrog algorithm was used to integrate the equations of motion, with an integration step of 1 fs. The average calculation frequency was 10 steps. The molecular dynamics calculation was followed by a second run of 2000 cycles of energy refinement until an average rms gradient of 0.06 kcal mol -1 Å-2 was achieved.

### Results

*NMR assignment and analysis*

The decamer is non-selfcomplementary, and the numbering scheme, which will be used, is the following:

$$5' - C_1\ C_2\ T_3\ C_4\ G_5{}^*\ C_6\ T_7\ C_8\ T_9\ C_{10} - 3'\ (I_M)$$
$$3' - G_{20}\ G_{19}\ A_{18}\ G_{17}\ C_{16}\ G_{15}\ A_{14}\ G_{13}\ A_{12}\ G_{11} - 5'\ (II)$$

2D-NOESY spectra of the native duplex in $H_2O$ were used to assign the resonances of all protons by standard DNA sequential assignment procedure (Fig. 1A). The proton chemical shifts were comparable with those reported for the same sequence by Leng et al. [22]. The NOESY spectra acquired for the *trans-EE* platinated duplex show connectivities for both strands and are quite similar to the corresponding spectra for the native duplex, except for the central part of the duplex (Fig. 1 A, B). The chemical shifts of the assigned resonances are listed in Table 1. The G5*-H8 resonance is shifted 0.17 ppm downfield relative to the unplatinated duplex. The H8 chemical shifts are, upon platination of the N7 atom, subject to several effects that can have equal or opposite signs. Usually, G-H8 downfield shifts are observed in the 0.5 - 1 ppm range for bifunctional platinum coordination as a combination of electron-withdrawing effect of the metal and destacking of the bases. Since several opposing effects may be involved a substantial downfield shift for H8 is not diagnostic for N7 platination. In a report describing a *cis*-DDP inter-strand cross-linked adduct of the present duplex the H8 resonances of the two platinated guanines are shifted 0.03 ppm and 0. 34 ppm, respectively, relative to the native duplex [22]. A comparison of the proton chemical shifts of the *trans-EE* and *cis*-DDP modified duplex shows remarkable similarities for the three end base pairs on each side of the duplex.

The most pronounced shift differences for the nonexchangeable protons involve the residues adjacent to G5, C4 and C6, and residue C16 complementary to G5. The imino protons experience relativly large downfield shifts at the terminal G-sites, 0.58 ppm (G11) and 0.35 ppm (G20), respectively. For the central GC base pairs minor downfield shifts are observed for the imino protons, except for the platinated G-residue which exhibit an upfield shift of -0.14 ppm. The imino protons involved in AT base pairing exhibit downfield shifts, 0.50 ppm (T3), 0.22 ppm (T7) and 0.18

ppm (T9), respectively. This variation induced by changes in ring current effects in the platinated duplex is difficult to relate to specific structural features.

The proton chemical shifts of the methyl and the methoxy resonances of the iminoether ligands, 2.58 ppm and 3.78 ppm, respectively, are almost identical to those of free *trans-EE* in solution [23]. In the NOESY spectra there are two significant crosspeaks observed between the duplex and the complex, namely between G5-H8 and the methoxy group and the methyl group, respectively. The corresponding G5-H8 ··· $CH_3O$ distance of 4.5 Å was calculated by the $r^{-6}$ relationship using the intensity of the integrated crosspeak of the intraresidue methyl - H6 distance

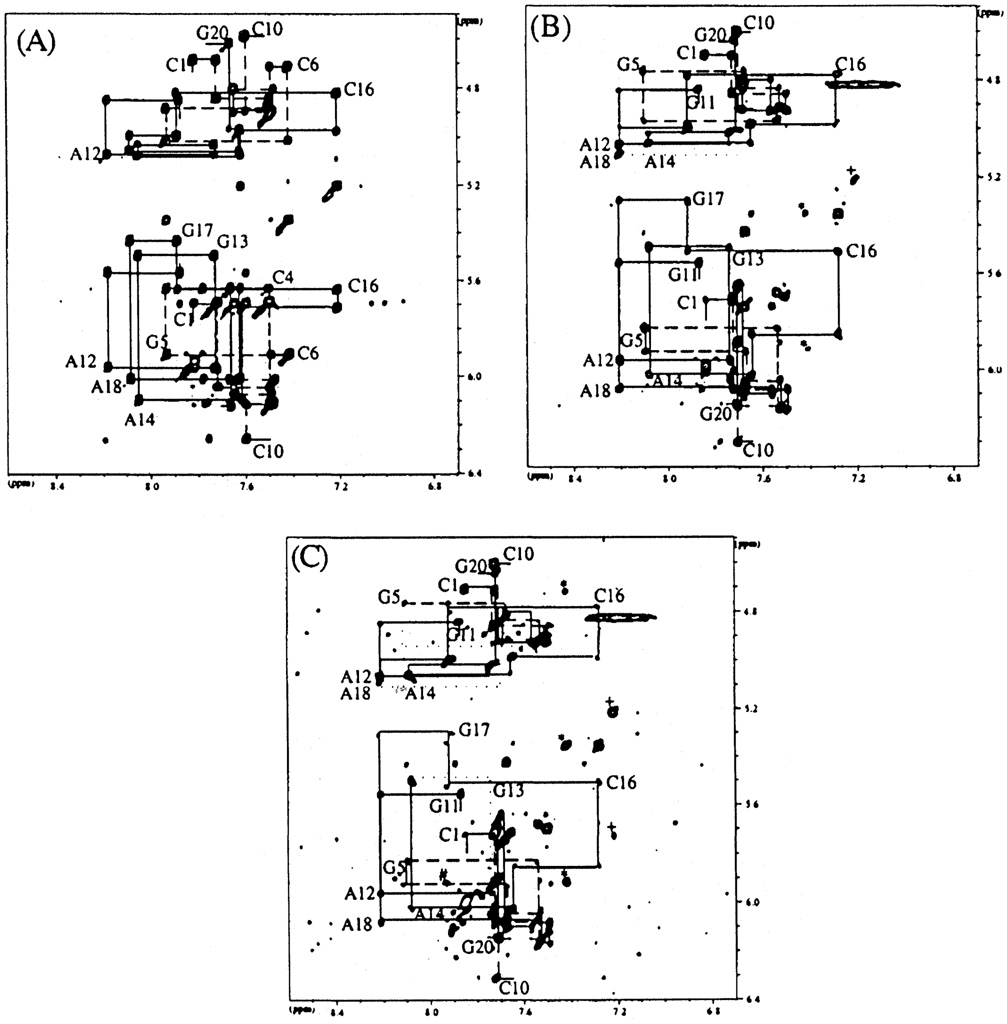

**Figure 1.** Contour plots of the resolution enhanced H8/6 - H1′/H5 and H3' regions in the 600 MHz NOESY spectra of the unplatinated duplex d(CCTCGCTCTC)·d(GAGAGCGAGG) (A) the *trans-EE* platinated duplex d(CCTCG*CTCTC)·d(GAGAGCGAGG) (B) and the platinated duplex on a later stage (C). In (B) and (C) crosspeaks of G5, C6 and C16 from the unplatinated duplex are marked #, * and + respectively. The sequential connectivities are indicated with broken lines for the upper strand, solid lines for the lower strand and dotted lines for the absent connectivities. The mixing time is 200 ms and the temperature 295 K.

of thymine T3 as a reference. This observation gives an approximate localization of the ligand relative to the duplex under the assumption that the iminoether ligands in the adduct retain the configurations as determined in the solid state [23].

The magnetic equivalence between the two symmetrically related iminoether ligands indicates a rapid exchange on the NMR time scale by rotation around the Pt-N7 and the Pt-iminoether bonds. In the final model structure the dihedral angle Cl-Pt-N/Pt-N-H was found to be

106°.The lack of bifunctional cross-linking by *trans-EE* may arises from steric crowding between the bulky carrier ligands and the nucleotide residues.

*Deplatination reactions*

In the NOESY spectra of the platinated duplex weak crosspeaks that could not be accounted for in the sequential assignment were observed at an early stage of the NMR investigation. This is not uncommon for platinated duplexes, where minor species may normally be present. However, during the next couple of weeks the intensity of these extra crosspeaks were found to gradually increase (Fig. 1 C) and a comparison with the NOESY map of the native duplex indicated that a reversible deplatination of the duplex had taken place. This was subsequently confirmed by an HPLC analysis of the sample several weeks later (Fig. 4) (*vide infra*). Evidently, the high NaCl concentration (0.2 M) used in the NMR sample to stabilize the duplex had promoted deplatination of G5*.

This unexpected result, showing that high NaCl concentration destabilizes the monofunctional Pt-N bond, may be relevant for other NMR studies of platinated oligonucleotides since it is common practice in this type of experiments to use high chloride concentration. Also in other studies involving high NaCl concentration (e.g. salt induced $B \rightarrow Z$ transition), the results should be regarded with caution if platinum complexes are involved.

*Molecular modeling*

To further probe the influence of *trans-EE* on the duplex geometry, molecular dynamic energy minimization calculations were carried out using the DISCOVER software [24]. In the molecular modeling approach the G5-H8 ···· $CH_3O$ (in the ligand) and G5-H8 ···· methyl (in the ligand) distances were set at 4.5 Å and 3 Å , respectively, the G5-N7···· Pt bond length at 2.0 Å, the Pt ···· Cl contact at 2.3 Å. Initially, the *trans-EE* complex was fixed in the geometry adopted in the solid state [23]. In the docking process the imino ligands were treated as rigid bodies and allowed free rotation around the Pt-N7 bond and the (imino)N-Pt-N(imino) axis, respectively. A stereo-view of the refined molecular model is shown in Fig. 2 and a close-up of the *trans-EE* - DNA environment is depicted in Fig. 3. The relevant contacts between the complex and the duplex are indicated by dotted lines.

Two of the contacts shown in figure 3 were used as NOE restraints during the molecular dynamics refinement. Due to partial overlap between the crosspeaks representing G5-H8 ···· methyl iminoether and G5-H8 ···· G5-H1′, respectively, the former distance is rather inaccurate. In the final model a stabilizing dipolar interaction is established between the carbonyl oxygen on G5 and the imino proton of *trans-EE*.

*HPLC analysis*

As mentioned above, a small fraction of the sample of the platinated duplex used for NMR experiments was analyzed by HPLC several weeks after its preparation. The duplex was now almost completely deplatinated as verified by the HPCL trace on the two native strands (I and II) (Fig. 4A,C). In addition, several new isomers were present representing, most likely, platinated species of the guanine rich lower strand. Evidently, the high NaCl concentration promotes displacement of platinum from the upper strand and its migration to the lower strand (at least in part), even at room temperature. In order to obtain more information on this apparently complex reaction pattern the platination reaction was repeated; the single (top) strand (I) with only one guanine (G5) was dissolved in acidic medium (pH 2.5, adjusted by $HClO_4$) and an excess of *trans-EE* was added as powdered solid. The reaction between single strand DNA (I) and *trans-EE* was then followed by HPLC at several time intervals for two days. After two hour more than half the amount of the oligonucleotide was found to be platinated as manifested by one major peak in the chromatographic trace ($I_M$). The platination reaction was completed after 18 hour; however, two additional species assignable to platinated single strands were detected by HPLC ($I_1$ and $I_2$). Apparently, a fraction of the first formed Pt-species is slowly transformed into two new species. After two days at room temperature, a 1:1 equilibrium is established between (at least) two platinated species. The species distribution curves for the reaction products are shown in Fig. 5. One may notice that for the platinated double-strand oligonucleotide the sample free of NaCl did not show sign of deplatination.

### Discussion

Previously we have reported on the biological activities of platinum-iminoether complexes and shown that the isomer *trans*-$[PtCl_2\{E\text{-}HN{=}C(OMe)Me\}_2]$ (*trans-EE*) possesses antileukaemic activity greater than that of the *cis* congener and comparable to that of cisplatin [3,6]. Furthermore, *trans-EE* did not exhibit cross-resistance with cisplatin in several human cancer cell lines [9], suggesting that the mechanism of action of *trans*-platinum iminoether complexes might be different from that of the *cis*-isomers.

**Table 1.** $^1$H NMR chemical shifts (ppm) for *trans-EE* platinated duplex: d(CCTCGCTCTC)·d(GAGAGCGAGG). The differences in chemical shifts between the platinated and unplatinated duplex (> 0.05 ppm) are given in parenthesis. All shifts are referenced to the HOD resonance at 4.81 ppm, T=295 K. Hb and Hf represent the bonded and the free amino protons.

| | H6/H8 | H2/H5 /$CH_3$ | H1' | H2' | H2" | H3' | H4' | Hb | Hf | H1/H3 |
|---|---|---|---|---|---|---|---|---|---|---|
| C1 | 7.84 | 5.98 | 5.71 | 2.32 | 2.58 (0.27) | 4.70 | 4.16 | 8.49 (0.08) | 7.16 (0.12) | |
| C2 | 7.73 | 5.71 | 6.08 | 2.21 | 2.59 | 4.86 | 4.26 | 8.46 | 7.12 (0.09) | |
| T3 | 7.50 | 1.66 | 6.17 (0.06) | 2.22 | 2.57 | 4.93 | 4.26 | | | 14.37 (0.50) |
| C4 | 7.54 | 5.68 | 5.83 (0.20) | 2.20 (0.09) | 2.69 (0.26) | 4.97 (0.11) | 4.26 (0.10) | 8.34 (-0.22) | 7.04 (0.11) | |
| G5 | 8.10 (0.17) | - | 5.92 | 2.59 (0.11) | 2.64 (0.08) | 4.77 (-0.25) | 4.13 (-0.28) | | | 12.79 (-0.14) |
| C6 | 7.68 (0.26) | 5.43 (0.08) | 6.09 | 2.21 (0.12) | 2.30 (-0.21) | 4.81 (0.09) | 4.29 (0.06) | 8.40 (0.27) | 6.79 (0.25) | |
| T7 | 7.56 (0.07) | 1.70 (0.09) | 6.10 | 2.31 | 2.62 | 4.93 | 4.25 | | | 14.05 (0.22) |
| C8 | 7.68 | 5.74 | 6.05 | 2.22 | 2.55 | 4.83 | 4.22 | 8.52 (0.08) | 7.22 (0.15) | |
| T9 | 7.53 | 1.78 | 6.16 | 2.24 | 2.55 | 4.92 | 4.22 | | | 14.11 (0.18) |
| C10 | 7.71 (0.11) | 5.88 (0.19) | 6.30 | 2.32 | 2.43 (0.12) | 4.60 | 4.07 | 8.30 (-0.10) | 7.36 (0.19) | |
| G11 | 7.87 | - | 5.56 | 2.47 | 2.66 | 4.84 | 4.17 | | | 12.95 (0.58) |
| A12 | 8.21 | 7.78 | 5.96 | 2.78 | 2.89 | 5.07 | 4.43 | | | |
| G13 | 7.74 | - | 5.50 | 2.60 | 2.71 | 5.02 | 4.39 | | | 12.77 (0.07) |
| A14 | 8.08 | | 6.02 (0.07) | 2.64 | 2.87 | 5.06 | 4.46 | | | |
| G15 | 7.65 | - | 5.85 (0.14) | 2.46 | 2.70 (0.07) | 4.98 | 4.41 | | | 12.80 |
| C16 | 7.28 (0.07) | 5.36 (0.15) | 5.51 (0.12) | 1.86 | 2.26 | 4.78 | 4.21 | 7.82 (-0.42) | 6.83 (0.56) | |
| G17 | 7.92 | - | 5.30 (0.13) | 2.69 | 2.75 | 5.00 | 4.32 | | | 12.97 (0.09) |
| A18 | 8.21 (0.12) | 7.86 | 6.08 (0.07) | 2.75 (0.11) | 2.92 (0.06) | 5.11 (0.06) | 4.47 | | | |
| G19 | 7.70 (0.08) | - | 5.64 | 2.54 | 2.68 | 5.01 | 4.38 | | | 13.00 |
| G20 | 7.71 | - | 6.13 | 2.38 | 2.46 | 4.64 | 4.24 | | | 12.72 (0.35) |

Coluccia *et al.* [3] have shown by replication mapping experiments that *trans-EE* blocks DNA polymerase at guanine residues in the context 5′-NGA (N = G or C) and thus exhibits sequence-selectivity similar to that observed for monofunctional binding to DNA duplexes of other transition metal complexes [25, 26]. In several studies of adducts between DNA and *cis-* or *trans-*DDP a series of complex isomerization reactions has been observed in which both intra- and inter-strand cross-links are produced. Interstrand cross-links are assumed to be the critical molecular lesions able to inactivate DNA as a template for replication [27, 28]. However, it has been shown that *trans-EE* has a greatly reduced ability to form such cross-links with respect to either *trans*-DDP

or *cis-EE* and *cis*-DDP [6]. Thus, the antitumor activity of *trans-EE* may originate from a different mode of action. In this respect the search for possible unpredictable interactions between the iminoether ligands and DNA may be of particular relevance.

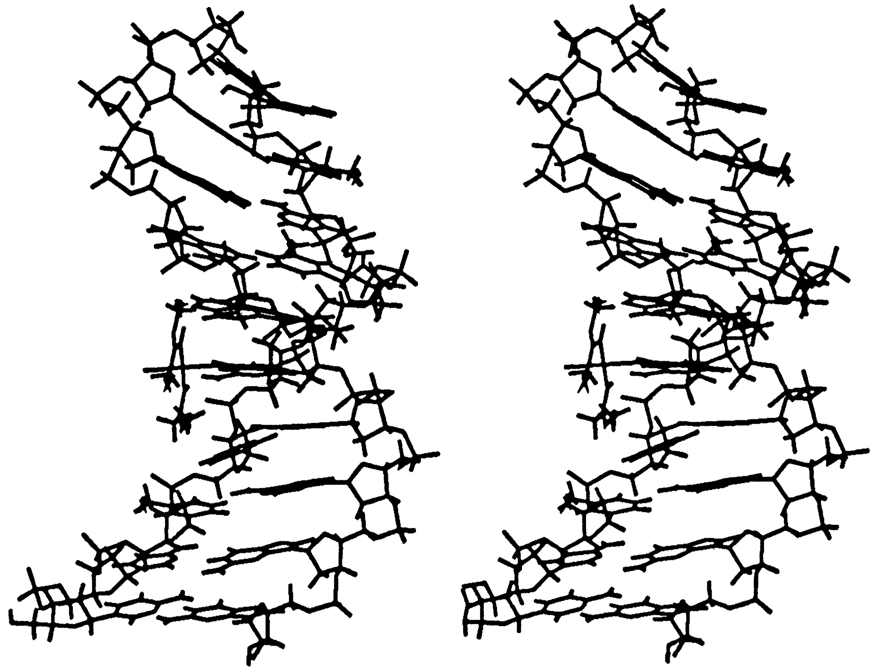

**Fig. 2**. Stereoview of the molecular model of the refined *trans-EE* monofunctional bound duplex d(CCTCG*CTCTC)·d(GAGAGCGAGG).

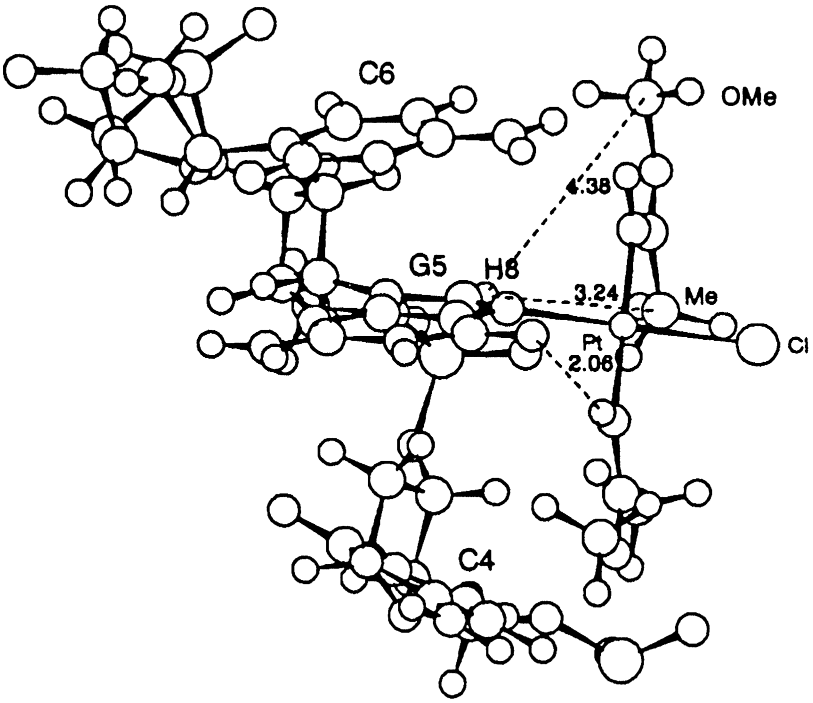

**Fig. 3.** Close-up model of the *trans-EE* – DNA environment.

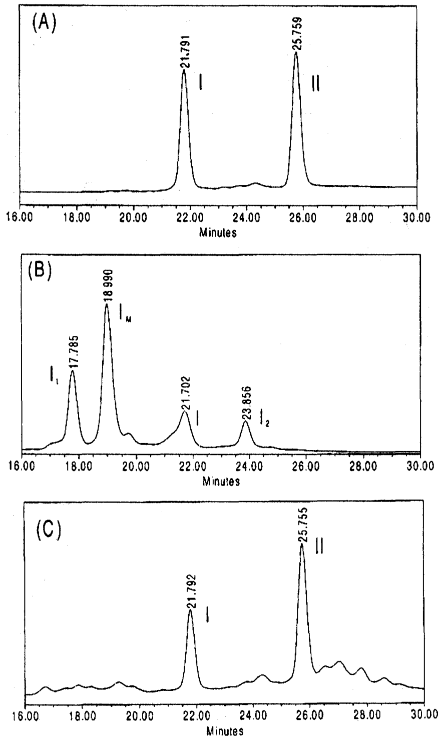

**Figure 4.** HPLC chromatograms of the complementary single strands I and II (A), trace of the species after *trans-EE* platination of single strand (I) (B), and a trace of the NMR sample a few weeks after the NOESY shown in Fig 1C, showing the unplatinated complementary single strands (I and II), using a MonoQ HR 10/10 (Pharmacia Biotech) column with a 0.3 to 1.0 M NaCl gradient, containing 10 mM NaOH.

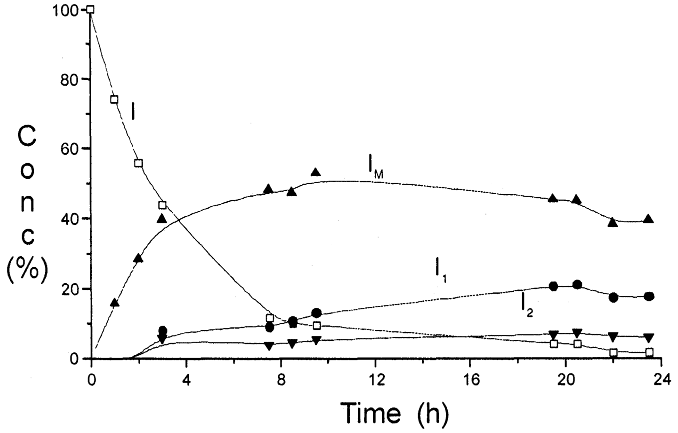

**Figure 5.** Species distribution curves for the increasing platinated species (▲ ($I_M$), ● ($I_1$), and ▼ ($I_2$)) and the decreasing amount of unplatinated oligonucleotide (I) at pH 2.5, no salt, at room temperature in $H_2O$.

The NMR data enabled us to localize the iminoethers relative to DNA, in a qualitative manner. The key ligand - DNA contact anchoring the iminoether to the duplex is manifested by two crosspeaks in the NOESY map involving G5-H8 and methoxy and methyl group of the iminoether ligands. The geometry optimization using the DISCOVER software resulted in a model where the iminoether ligand is located relative to the duplex in an energetically favorable position, *e.g.* the guanine carbonyl G5-O is reasonably close to the imino proton of the second iminoether ligand. However, the G5-O ···· H-N contact mentioned above does not represent a regular H-bond, the angle O-H-N being close to 90°. The hydrogen bonding ability of the amine ligand has been proposed to be of relevance to the cytotoxic efficacy of cisplatin.

From the model described above it is evident that the bulky carrier ligands of *trans-EE* effectively prevent formation of bifunctional adducts of the type observed for the analogous *trans*-DDP and also for *cis*-DDP. Thus, the intra- and inter-strand cross-linking lesions of DNA induced by *cis*-DDP and assumed to be important for its antitumor efficacy, are not the critical lesions for *trans*-EE. Other platinum complexes, *e.g.* $[Pt(dien)Cl]^+$, form monofunctional lesions with DNA, but they exhibit no antitumor activity. On the other hand, a group of monofunctionally DNA binding platinum complexes containing aromatic amine ligands were reported time ago to exhibit antitumor activity. The mechanism for this activity was suggested to involve intercalation of the planar aromatic ligand in DNA [29]. A scheme requiring intercalation does not appear likely for the iminoether ligand. However from our model it is possible to estimate the bending the oligonucleotide undergoes as a consequence of monofunctional platination by *trans-EE* at G5. Excluding the first and the last base-pairs which are expected to be rather mobile, the helical axes calculated for the upper and lower triplet of base pairs make an angle of ca. 45°. This value also corresponds to the dihedral angle between base-pairs of the upper portion of the oligo and base pairs of the lower portion (average value ca. 50°). Such a bending angle is comparable to those observed for bifunctional adducts of cisplatin of both intra-strand and inter-strand types. Moreover, the bending is towards the minor groove as found in cisplatin inter-strand cross-link [30-32] and not towards the major groove as found for cisplatin intra-strand cross-link [33-36]. This result was rather unexpected and we suspect it is the rigidity and planarity of the carrier ligand to be responsible for it. Although warrant other confirmations, this large bending of the DNA could apply also to other platinum complexes with large planar carrier ligands and could be responsible for the high toxicity (and in some cases also antitumour activity) observed for some of these complexes [37, 38].

**Acknowledgements**. Financial support by the European Commission BIOMEDII program (Contract BMH4-CT97-2485) and COST action D8/007/97 and D8/012/97 are gratefully acknowledged. Thanks are extended to the Norwegian Research Council for a fellowship to B.A. We thank Dr. J. Arpalahti, University of Turku, for providing additional HPLC and electrophoretic analysis.

**References**

1. B. Lippert (ed.) *Cisplatin. Chemistry and Biochemistry of a Leading Anticancer Drug*. Wiley-VCH, Zurich, 1999.
2. G. Natile, M. Coluccia, in: *Topics in Biological Inorganic Chemistry, Metallopharmaceuticals I, DNA Interactions*. **1**, 73-98, (1999) (M. J. Clarke, P. J. Sadler, eds). Springer-Verlag, Berlin.
3. M. Coluccia, A. Nassi, F. Loseto, A. Boccarelli, M.A. Mariggio, D. Giordano, F.P. Intini, P. Caputo, G. Natile. *J. Med. Chem.* (1993) **36**, 510-512.
4. N. Farrell, in: *Metal Ions in Biological Systems*. A. Sigel and H. Sigel (eds.) (1996) **32**, 603-639. Marcel Dekker, Inc., New York. N. Farrell, L.R. Kelland, J.D. Roberts, M. Van Beusichem. *Cancer Res.* (1992) **52**, 5065-5072.
5. L.R. Kelland, C.F.J. Barnard, I.G. Evans, B.A. Murrer, B.R.C. Theobald, Wyer, S.B., P.M. Goddard, M. Jones, M. Valenti, A. Bryant, P.M. Rogers, K.R. Harrap. *J. Med. Chem.* (1995) **38**, 3016-3024.
6. M. Coluccia, A. Boccarelli, M.A. Mariggio, N. Cardellicchio, P. Caputo, F.P. Intini, G. Natile. *Chem. Biolog. Interactions,* (1995), **98**, 251-266.
7. R. Zaludova, A. Zakovska, J. Kasparkova, Z. Balcarova, O. Vrana, M. Coluccia, G. Natile, V. Brabec. *Mol. Pharmacol.* (1997) **52**, 354-361.
8. V. Brabec, O. Vrana, O. Novakova, V. Kleinwachter, F.P. Intini, M. Coluccia, G. Natile. *Nucleic Acids Res.* (1996) **24**, 336-341.
9. M. Coluccia, A. Nassi, A. Boccarelli, D. Giordano, N. Cardellicchio, F.P. Intini, G. Natile, A. Barletta, A. Paradiso. *International J. of Oncology* (1999) **15** in press.
10. A. Boccarelli, M. Coluccia, F.P. Intini, G. Natile, D. Locker, M. Leng. *Anti-Cancer Drug Design* (1999), **14**, 253-264.
11. R. Zaludova, G. Natile, V. Brabec. *Anti-Cancer Drug Design* (1997), **12**, 295-309.
12. B. Malfoy, B. Hartmann, M. Leng, *Nucleic Acid Res.*, (1981), **9**, 5659-5669.
13. F. Legendre, J. Kozelka, J.–C. Chottard. *Inorg. Chem.* (1998), **37**, 3964-3967.
14. A.M. Maxam, W. Gilbert. *Proc. Natl. Acad. Sci. USA* (1977) **74**, 560-564.

15. A. Eastman, M.A. Barry, M.A. *Biochemistry* (1987) **26**, 3303-3307.
16. V. Sklenar, M. Piotto, R. Leppik, V. Saudek. *J. Magn. Reson. A* (1993) **102**, 241-245.
17. D.J. States, R.A. Haberkorn, D.J. Ruben. *J. Magn. Reson.* (1982) **48**, 286-292.
18. J.J. Led, H. Gesmar. *Chem. Rev.* (1991) **91**, 1413-1426.
19. FELIX (version 2.30) Processing package, BIOSYM/MSI.
20. Bruker 1D WIN-NMR (version 960901.2) and 2D WIN-NMR (version 6.02).
21. Waters Millenium 32 software.
22. F. Paquet, C. Perez, M. Leng, G. Lancelot, J.–M. Malinge. *J. Biomol. Struct. Dynam.* (1996) **14**, 67-77.
23. R. Cini, P.A. Caputo, F.P. Intini, G. Natile. *Inorg. Chem.* (1995) **34**, 1130-1137.
24. BIOSYM User Guide for Insight II (version 2.3.5), Discover (version 2.9.5) and NMR Refine (version 2.3).
25. E. Sletten, N.Å. Frøystein, in: *Metal Ions in Biological Systems.* A. Sigel and H. Sigel (eds.) **32**, 397-418. Marcel Dekker, Inc.: New York (1996).
26. E. Molderheim, B. Andersen, N.Å. Frøystein, E. Sletten. *Inorg. Chim. Acta,* (1998) **273**, 41-46.
27. G. Laurent, L.C. Erickson, N.A. Sharkey, K.W. Kohn. *Cancer Res.*, (1981) **41**, 3347-3351.
28. J.J. Roberts, R.J. Knox, M.F. Pera, F. Friedlos, D.A. Lydall in: *Platinum and Other Metal Coordination Compounds in Cancer Chemotherapy.* M. Nicolini (Ed.) 16-31. Martinus Nijoff Publishing, Boston (1988).
29. L. S. Hollis, W. I. Sundquist, J. N. Burstyn, W. J. Heiger-Bernays, S. F. Bellon, K. J. Ahmed, A. R. Amundsen, E. W. Stern, S. J. Lippard. *Cancer Res.*, (1991) **51**, 1866-1875.
30. H. Huang, L. Zhu, B. R. Reid, G. P. Drobny, P. B. Hopkins. *Science* (1995) **270**, 1842-1845.
31. F. Paquet, C. Pérez, M. Leng, G. Lancelot, J.-M. Malinge. *J. Biomol. Struct. Dyn.* (1996) **14**, 67-77.
32. F. Coste, J.–M. Malinge, L. Serre, W. Shepard, M. Roth, M. Leng, C. Zelwer. *Nucleic Acid Res.* (1999) **27**, 1837-1846.
33. P. Takahara, A. C. Rosenzweig, C. A. Frederick, S. J. Lippard. *Nature* (1995) **377**, 649-652.
34. P. Takahara, C. A. Frederick, S. J. Lippard. *J. Am. Chem. Soc.* (1996) **118**, 12309-12321.
35. A. Gelasco, S. J. Lippard. *Biochemistry* (1998) **37**, 9230-9239.
36. D. Yang, S. S. G. E. van Boom, J. Reedijk, J. H. van Boom, A. H.–J. Wang, *Biohemistry* (1995) **34**, 12912-12920.
37. N. Farrell, T.T.B. Ha, J. -P. Souchard, F. L. Wimmer, S. Cros, N. P. Johnson. *J. Med. Chem.* (1989) **32,** 2240-2241.
38. N. Farrell, L. R. Kelland, J. D. Roberts, M. Van Beusichem *Cancer Res.* (1992) **52,** 5065-5072.

**Received: November 29, 1999 - Accepted: November 25, 1999 - Received in revised camera-ready format: December 13, 1999**

